# Evaluating the possibility of detecting evidence of positive selection across Asia with sparse genotype data from the HUGO Pan-Asian SNP Consortium

**DOI:** 10.1186/1471-2164-15-332

**Published:** 2014-05-02

**Authors:** Xuanyao Liu, Woei-Yuh Saw, Mohammad Ali, Rick Twee-Hee Ong, Yik-Ying Teo

**Affiliations:** Saw Swee Hock School of Public Health, National University of Singapore, MD3 16 Medical Drive, Singapore, 117597 Singapore; NUS Graduate School for Integrative Science and Engineering, National University of Singapore, Singapore, 117456 Singapore; Life Sciences Institute, National University of Singapore, Singapore, 117456 Singapore; Department of Statistics and Applied Probability, National University of Singapore, Singapore, 117546 Singapore; Agency for Science, Technology and Research, Genome Institute of Singapore, Singapore, 138672 Singapore

**Keywords:** Haplotype phasing, Positive selection, Population structure, Genetic diversity

## Abstract

**Background:**

The HUGO Pan-Asian SNP Consortium (PASNP) has generated a genetic resource of almost 55,000 autosomal single nucleotide polymorphisms (SNPs) across more than 1,800 individuals from 73 urban and indigenous populations in Asia. This has offered valuable insights into the correlation between the genetic ancestry of these populations with major linguistic systems and geography. Here, we attempt to understand whether adaptation to local climate, diet and environment partly explains the genetic variation present in these populations by investigating the genomic signatures of positive selection.

**Results:**

To evaluate the impact to the selection analyses due to the considerably lower SNP density as compared to other population genetics resources such as the International HapMap Project (HapMap) or the Singapore Genome Variation Project, we evaluated the extent of haplotype phasing switch errors and the consistency of selection signals from three haplotype-based approaches (iHS, XP-EHH, haploPS) when the HapMap data is thinned to a similar density as PASNP. We subsequently applied haploPS to detect and characterize positive selection in the PASNP populations, identifying 59 genomics regions that were selected in at least one PASNP populations. A cluster analysis on the basis of these 59 signals showed that indigenous populations such as the Negrito from Malaysia and Philippines, the China Hmong, and the Taiwan Ami and Atayal shared more of these signals. We also reported evidence of a positive selection signal encompassing the beta globin gene in the Taiwan Ami and Atayal that was distinct from the signal in the HapMap Africans, suggesting the possibility of convergent evolution at this locus due to malarial selection.

**Conclusions:**

We established that the lower SNP content of the PASNP data conferred weaker ability to detect signatures of positive selection, but the availability of the new approach haploPS retained modest power. Out of all the populations in PASNP, we identified only 59 signals, suggesting a strong need for high-density population-level genotyping data or sequencing data in order to achieve a comprehensive survey of positive selection in Asian populations.

**Electronic supplementary material:**

The online version of this article (doi:10.1186/1471-2164-15-332) contains supplementary material, which is available to authorized users.

## Background

Asia is the largest continent on Earth, covering 30% of the available land area and playing host to more than 60% of the human populations in the world. With a latitudinal range of 11.6°S to 81.9°N and a longitudinal range of 27.3°E to 169.0°W, Asia possesses extremely diverse climates and geographical conditions, with temperatures ranging from arctic in northern Asia to tropical along the Equator and humidity ranging between below 10% in the interior of the continent to in excess of 90% along the coast and in Southeast Asia. Comprising 49 countries, many of which contain a wide variety of ethnicities and subpopulations, Asia also hosts a myriad of unrelated language families such as the Sino-Tibetan languages predominantly spoken in East Asia; the Indo-European and Dravidian languages predominantly spoken in south and central Asia; the Altaic languages predominantly found in Korea, Japan, and central and northern Asia; and the Austronesian and Tai-Kadai languages commonly spoken in Southeast Asia.

The distinct languages in Asia have limited the extent of historical interactions between different population groups, leading to a greater degree of genetic homogeneity between populations sharing the same linguistic system while extending the genetic differences between populations with different linguistic systems. This situation is similar to that present in the Africa continent [1]. The diverse geographical and climatic conditions have directly influenced the rate of population growth and movement, as well as urbanization and agricultural land use in different parts of Asia, where differential sanitation and health systems have exerted profound influence on the burdens of diseases in different parts of Asia, particularly those of vector-borne infectious diseases such as malaria and dengue [2].

The availability of genetic datasets for global populations from the International HapMap Project (HapMap) [3–5], the Human Genome Diversity Project (HGDP) [6] and the Singapore Genome Variation Project [7] have facilitated research into how humans have adapted differentially to the climate of their habitats [8–10], prevalent diet [11, 12] and other environmental assaults including those from pathogens and allergens [13–17]. Several reports have also described the convergent evolution of hemoglobin genes in populations residing in different high-altitude locations around the world, allowing humans to adapt to an environment with reduced levels of oxygen [18–22]. Given the diversity in geography, culture, environment and language that is present in Asia, there have not been many systematic reports investigating the genomic evidence of local adaptations of Asian populations, especially those of the ethnic minorities and indigenous populations [8, 10, 13, 23, 24].

The HUGO Pan-Asian SNP Consortium (PASNP) was a collaborative agreement established to perform an unprecedented genetic survey of Asian populations, and has provided a valuable resource of around 54,974 autosomal single nucleotide polymorphisms (SNPs) for 1,928 individuals from 73 Asian and two non-Asian HapMap populations (Europeans: CEU, and Nigerian Africans: YRI) [25]. The individuals surveyed in PASNP not only included those from urban populations, but also from the indigenous populations and ethnic minorities. Due to the sparse density of SNPs across the genome, surveys into genomic evidence of local adaption in this dataset have depended on SNP-based methods such as the Wright F_ST_ index that highlights striking differences in allele frequencies across a region [24], instead of haplotype-based approaches such as the iHS [8] or XP-EHH [13] that confer higher statistical power. The assumption behind both allele frequency and haplotype-based methods is the same: the frequency of the beneficial allele in a population will rise uncharacteristically rapidly, such that (1) the variants from neighboring SNPs that reside on the same haplotype as the beneficial allele will be co-inherited and there is insufficient time for recombination events to break down this extended haplotype; and (2) the beneficial allele and these variants will be found at notably higher frequencies in this population, than in other populations not experiencing the same evolutionary pressure to adapt.

The introduction of a new haplotype-based approach haploPS [23] presents the opportunity to perform a systematic survey and characterization of genomic signatures of local adaptation in the PASNP dataset. As haploPS relies on locating the extended haplotype form that the advantageous allele resides on, the expectation is the sparser SNP density from PASNP will still allow sufficient number of SNPs to anchor an extended haplotype. Comparing the selected haplotypes from different populations will also allow the inference of whether the signals across different populations are attributed to the same evolutionary event, or whether they are independent and are the consequence of convergent evolution.

Here, we aim to discover and characterize the origin and segregation of positive selection signals that are present in the Asian populations in PASNP. As the ability to detect the extended haplotypes using haplotype-based approaches relies on the accuracy and fidelity of the haplotype phasing, we first compared the extent of switch errors found in phasing the haplotypes for the full set of SNPs from the three population panels in Phase 2 of the HapMap (HapMap2), to the extent of the errors present when the SNP density for the HapMap samples has been reduced to a similar content as the PASNP data. In addition, we evaluated the power of haploPS via simulations to locate true signals of positive selection for haplotype data with a sparse set of SNPs, and also performed an empirical comparison of the degree of overlap in the selection signals found for the HapMap populations using the full and sparse sets of SNPs. The latter two exercises will allow an evaluation of the degree of power loss as a result of the sparser SNP density. By clustering the 73 PASNP populations into 31 groups according to shared linguistic systems and geographical proximity of the populations, we run haploPS to discover evidence of local adaptations in these 31 groups. The results provide the first systematic and large-scale survey of local adaptation in Asia, particularly in mapping the genomic features for the indigenous populations that are attributed to evolutionary pressures.

## Results

### Population structure analyses

**Figure 1:**
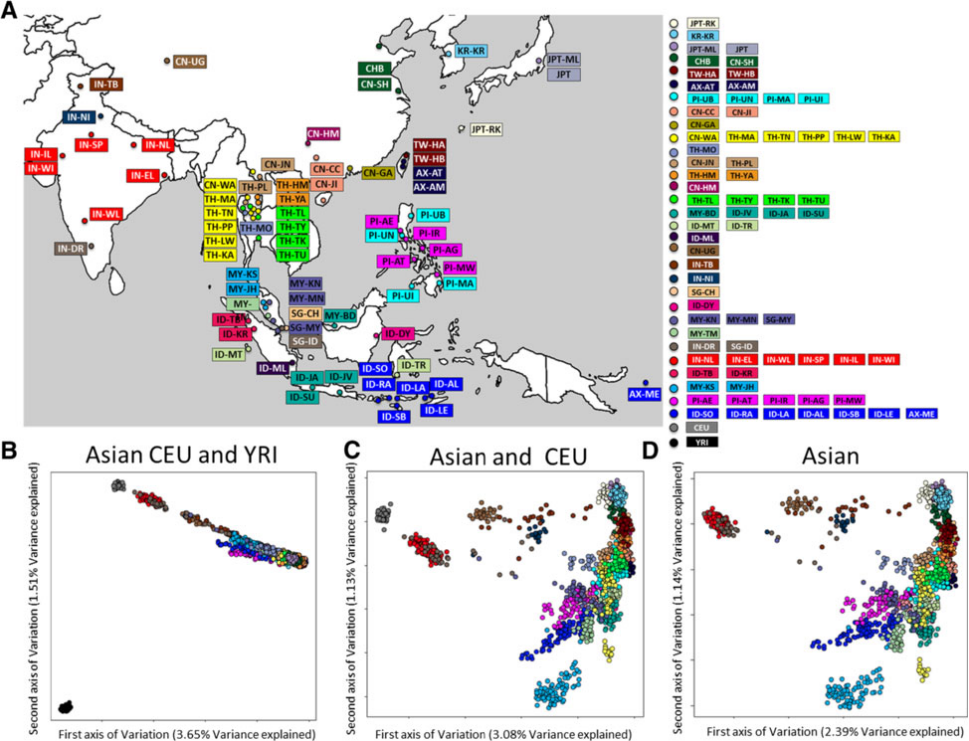
**Distribution and principal component analyses of the populations in the HUGO Pan-Asian SNP Consortium (PASNP). (A)** Geographical map indicating the locations of the populations in PASNP with the colors of the population labels determined by genetic, linguistic and geographic similarities of the populations. Three sets of principal components analyses were performed between: **(B)** the PASNP populations with HapMap Europeans (CEU) and Africans (YRI); **(C)** the PASNP populations and CEU; and **(D)** the PASNP populations only. Each circle represents an individual from the population grouping indicated by the assigned color, and the population labels correspond to the original labels used by PASNP.

**Figure 2:**
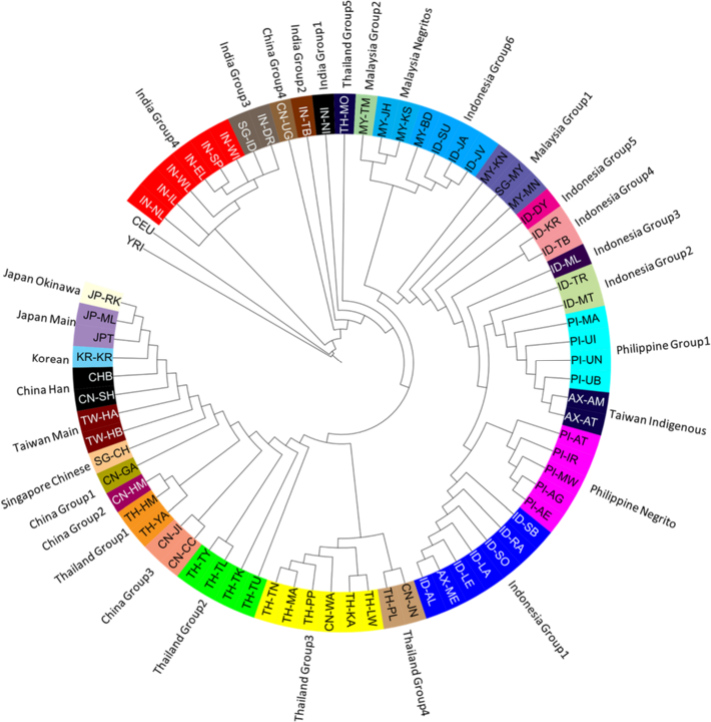
**Clustering of the PASNP and HapMap populations.** A phylogenetic tree obtained using a maximum likelihood procedure in the PHYLIP package on the genotype data for SNPs in the autosomal chromosomes to cluster the PASNP and HapMap populations. Cross-referencing the populations found within the same major branches indicated that genetic similarities concurred with linguistic and geographic similarities.

The PASNP dataset consists of genotypes at 54,974 autosomal SNPs for 1,928 individuals from 73 Asian and two non-Asian HapMap populations (CEU, YRI; Figure 1A), and we followed the definitions of the populations as introduced by PASNP ([25], Additional file 1: Table S1). Principal component analyses (PCA) of all 75 populations indicated that the Asian populations were genetically distinct from the Africans, and populations of South Asian ancestry were closer to the Europeans than other Asian populations (Figure 1B). The East and Southeast Asian populations were generally clustered together when analysed together with the Africans and Europeans, although Ladakhi Indians (IN-TB) and Uyghur Chinese (CN-UG) appeared to be between the South Asians and East/Southeast Asians. Further analyses without the African samples offered greater resolution to the degree of genetic homogeneity between East and Southeast Asian populations, regardless of whether the Europeans were included (Figure 1C) or excluded (Figure 1D). Insights from the PCA into the genetic similarity of the Asian populations concurred with those offered by a phylogenetic tree constructed with the maximum likelihood-based PHYLIP software, indicating that the Asian populations are clustered according to shared linguistics and geographical proximity of the populations (Figure 2). The positive selection analyses were thus performed on 31 groupings of the 73 Asian populations, where the groupings were determined on the basis of linguistic similarities and geographical closeness of the populations.

### Inference of haplotype phasing accuracy with sparse genotype data

The application of haplotype-based methods to locate genomic signatures of positive selection requires the PASNP genotype data to be phased. However, due to the sparse genotype density with around 50,000 SNPs across the autosomal chromosomes, we investigated using the HapMap2 populations whether the SNP density of PASNP will affect the accuracy of the phasing. By taking the haplotypes available from the HapMap resource as the benchmark since they have been phased using PHASE and incorporated pedigree information for the CEU and YRI trios, we estimated the extent of switch errors when the unrelated samples from HapMap2 were phased using SHAPEIT [26] with the full set of SNPs and when the SNP density was reduced to that of PASNP. We observed that there were considerably higher switch errors for all three population panels (Table 1). For the East Asian panel, the switch error rate increased from 1.5% to 12.5%. The higher switch errors will likely result in lower statistical power for haplotype-based approaches to detected extended haplotypes, since such methods rely explicitly on modeling or locating long stretches of haplotypes against the recombination background.

**Table 1:** **Switch error rates in phasing the HapMap2 samples with SHAPEIT on the original 2.6 million SNPs and on a reduced panel of around 55,000 SNPs**

HapMap2 panel	Switch error rates	
	Original density	Reduced density
**JPT + CHB**	1.5%	12.5%
**CEU**	1.0%	9.6%
**YRI**	5.1%	10.2%

### Power of positive selection methods with haplotypes from sparse genotype data

**Figure 3:**
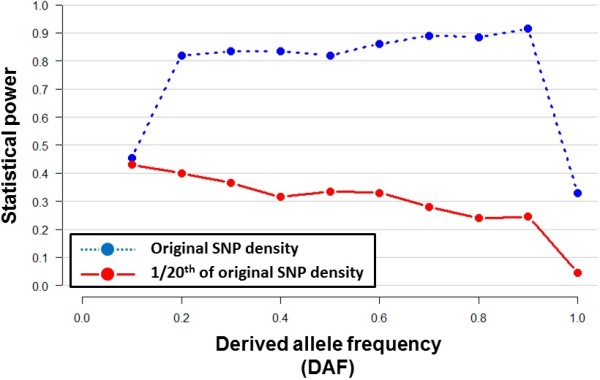
**Statistical power of haploPS.** Statistical power of haploPS to successfully identify a genomic region simulated to possess an advantageous derived allele at different allele frequency was evaluated in two settings using simulated data that is publicly available from the haploPS website: (i) with data of the original SNP density (blue dotted line and circles); and (ii) when the SNP density is reduced to 1/20^th^ of the original SNP density which is meant to reflect the density of SNPs in PASNP (red solid line and circles). Power was calculated from 2,000 simulated regions at a false discovery rate of 1%, defined against the empirical null distribution of the haploPS score obtained from a separate set of 2,000 simulated regions without positive selection.

We performed a simulation study to investigate the statistical power of haploPS to detect positive selection in the human genome. This used the publicly available simulated data from the haploPS resource, where 2,000 genomic regions each carrying a positively selected allele and SNPs at a density similar to the HapMap2 dataset (around 20 SNPs per 10 kb) were generated. We calculated the power of haploPS to detect the positively selected regions using the original datasets and when the datasets were thinned to a SNP density that was similar to the PASNP (around 1 SNP per 10 kb). We observed that the power of haploPS to locate the selection signals was much reduced for the thinned datasets as compared to the original datasets, with almost no power to locate a signal that was at fixation (Figure 3). For example, at a derived allele frequency of 90%, a higher SNP density conferred a 91% power whereas the reduced density data only offered a power of 24%. Statistical power was higher for low derived allele frequencies, presumably because these signals would have spanned a longer genetic distance and thus included sufficient number of SNPs to anchor the signal.

We also performed an empirical comparison of the consistency in the positive selection signals that have been detected by haploPS, iHS, XP-EHH and Fst for HapMap2 populations with the original and thinned datasets. The purpose of this was to investigate the extent of the positive selection signals that were present in the analyses on the reduced-density data with around 55,000 SNPs, compared to what were originally detected in the full HapMap2 dataset with 2.6 million SNPs. More importantly, it was used to benchmark the consistency in the signals that were present in both datasets. All four methods managed to identify signals in the reduced dataset, although haploPS only managed to identify three regions from the HapMap2 populations, while 57 and 9 regions were deemed to exhibit significant evidence of positive selection by iHS and XP-EHH respectively; and Fst discovered 123 selection regions, which is similar to the number of regions (159) detected in the original dataset (Table 2). However, of the three signals identified by haploPS, two were similarly present when the analysis was performed on the full HapMap2 dataset. For iHS, six of the 57 signals overlapped with the full dataset analysis;none of the nine regions overlapped for XP-EHH; and 28 out of 123 signals overlapped for Fst analysis. These findings suggest that haploPS minimized the extent of inconsistent discoveries with datasets of differing SNP densities.

**Table 2:** **Empirical comparison of consistency in positive selection signals in HapMap2 panels using haploPS, iHS and XP-EHH on the original 2.6 million SNPs and on a reduced panel of around 55,000 SNPs**

Method	Number of positively selected region identified	
	Original density	Reduced density (overlapped with original^1^)
**haploPS**	310	3 (2)
**iHS**	188	57 (6)
**XP-EHH**	35	9 (0)
**Fst**	159	123 (28)

^1^Number of signals that have been identified by both the original-density dataset and the reduced-density dataset.

### Positive selection in the PASNP data

**Figure 4:**
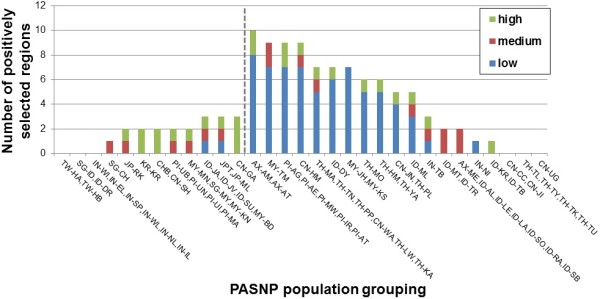
**Frequency spectrum of positive selection regions in PASNP.** Summary of the number of positive selection signals in each of the 31 PASNP population groupings, classified according to the inferred frequencies of the advantageous alleles in three categories: (i) high, where derived allele frequency (DAF) ≤ 30%; (ii) medium, 30% < DAF < 80%; and (iii) high, DAF ≥ 80%. The vertical dashed line separates the urban and cosmopolitan populations (left of line) from the ethnic minorities and indigenous populations (right of line).

The PASNP genotype data of 54,974 autosomal SNPs were phased with SHAPEIT using reference haplotypes from Phase 1 of the 1000 Genomes Project as a scaffold, and the haplotypes from each of the 31 PASNP population groupings were analyzed with haploPS for evidence of positive selection. A total of 59 genomic regions were identified to be positively selected (Additional file 1: Table S2), of which 25 regions were present in at least two groups and there were more signals present in the ethnic minorities and indigenous populations as compared to the urban cosmopolitan populations. Inference on the frequencies of the variants that were positively selected suggested that urban cosmopolitan populations tend to carry signals in the medium frequency range (between 30% and 80%) and in the high frequency range (>80%), whereas indigenous populations tend to carry signals that were present at low frequencies (<30%) in the populations (Figure 4).

**Figure 5:**
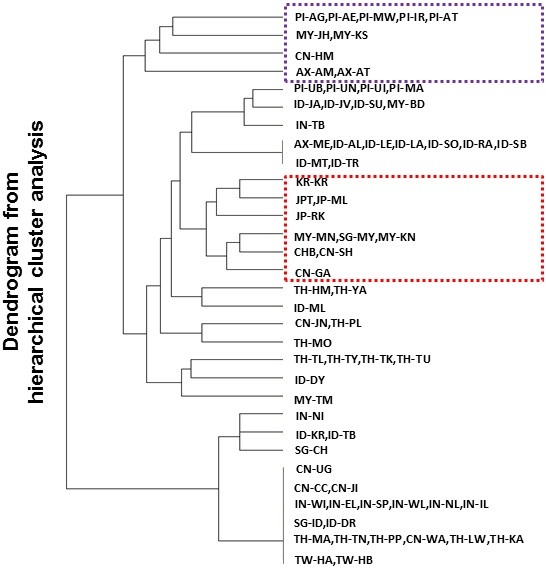
**Clustering of PASNP population groups by selection signals.** Hierarchical clustering of the 31 PASNP population groups according to the absence or presence of the 59 positive selection signals that have been identified by haploPS. Each of the 59 signals is present in at least one of the 31 population groups. The hierarchical clustering is performed using the Ward’s minimum variance method with the *hclust* command in R. Populations found in one of the major branches correspond to indigenous populations from Malaysia, Philippines, Thailand and China (upper purple box), while populations found in one of the sub-branches correspond to those in northern East Asia (lower red box).

We derived a similarity matrix for the 31 population groupings by querying the extent of sharing across the 59 positively selected regions, and a hierarchical cluster analysis on this matrix yielded a major branch that consisted of four indigenous population groups: Malaysia (Negrito), Philippines (Negrito), China Hmong and Taiwan Ami and Atayal (Figure 5). There were clear geographical delineations in the clustering, where the northern East Asian populations such as the Japanese, Koreans, and the northern Han Chinese were found in one of the sub-branches, and where the Thailand and Indonesian populations were more commonly clustered together.

**Figure 6:**
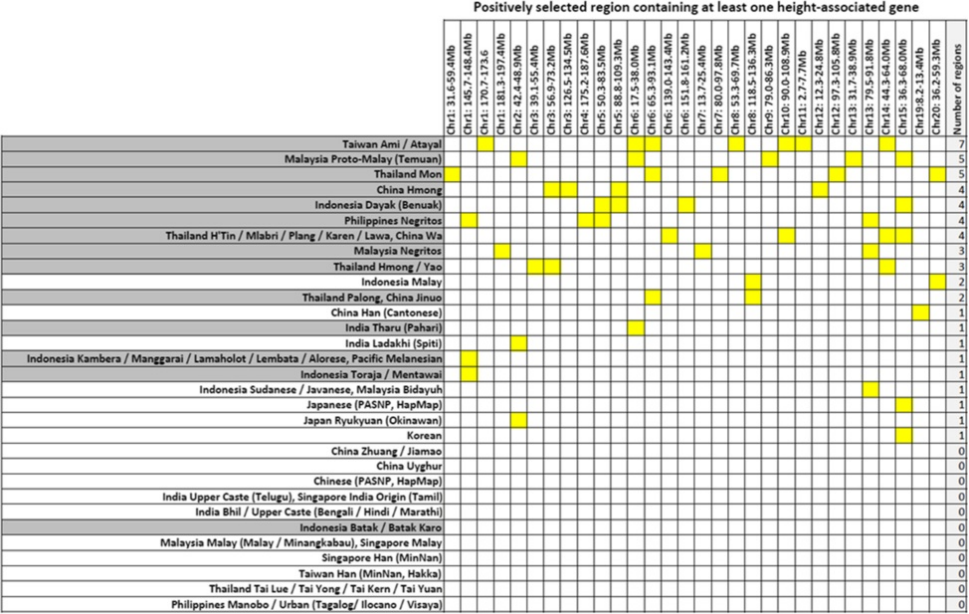
**Selection signals containing at least one height-associated gene.** Distribution of the 30 positive selection signals that spanned at least one height-associated gene across the 31 PASNP population groupings. Each yellow block indicates that the specific selection signal (column header) is present in a particular group of PASNP populations (row header). The groups are ranked according to the number of selection signals in descending order (last column). Population groupings that correspond to indigenous populations are shaded in grey.

We observed that 30 of the 59 regions encompassed genes that have been reported to be associated with human height, which corresponded to significant evidence of over-representation even after accounting for the greater proportion of height genes that have been reported (Binomial test of over-representation p-value = 9.98 × 10^−5^, see Additional file 1: Methods). The distribution of these 30 regions was primarily present in the indigenous populations from Malaysia (Negrito), Philippines (Negrito), Taiwan (Ami and Atayal) and Thailand (Mon, Hmong, Yao, H’Tin, Mlabri, Plang, Karen, Lawa and Palong), and not commonly found in urban populations or in HapMap CEU or YRI (Figure 6).

A region on chromosome 2 between 196.8 Mb and 198.0 Mb was identified in 12 of the 31 population groupings (Additional file 1: Table S2), and encompassed selection signals located between 197.0 Mb and 197.5 Mb previously reported in nine populations from HapMap and SGVP [23] that spanned *PGAP1*, a gene which caused perinatal lethality and male infertility in mice [27]. This region similarly exhibited consistent evidence from iHS and XP-EHH analyses in HGDP populations from Europe, East Asia and South Asia (Additional file 1: Figure S1). The 12 extended haplotype forms from the population groupings were perfectly identical (Additional file 1: Figure S2), yielding a haplotype similarity index (HSI) of 1.00. We adopted the interpretation of the HSI as suggested by the simulation results on the sensitivity and specificity from a previous study [23]: a high HSI (defined as ≥ 0.98) means the extended haplotype forms from the different populations are highly similar and are thus likely to be carrying the same selected allele from a single mutation event; a low HSI (defined as ≤ 0.9) suggests that the haplotype forms are considerably different and the selection signals are likely to be independent and indicative of convergent evolution. The HSI of 1.00 thus suggests that the same advantageous mutation is likely to be responsible for the selection signals present in the 12 population groupings, and this mutation has emerged prior to the divergence of these populations.

**Figure 7:**
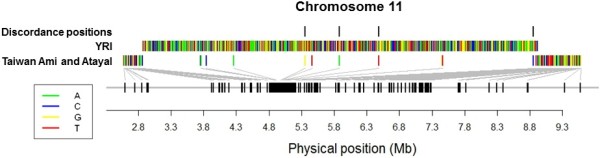
**Selected haplotype forms at** ***HBB*** **in YRI and Taiwan aborigines.** HaploPS identified the extended haplotypes that presented evidence of positive selection at *HBB* in HapMap Nigerians (YRI) and in two Taiwan indigenous populations, the Ami and Atayal, which were both found at 10% in the respective populations. The selection signals most likely stem from different mutation events in light of the low haplotype similarity index (HSI) of 0.63 between the haplotype forms from YRI and Taiwan.

One of the 59 regions included an extended haplotype of almost 5 Mb on chromosome 11 (between 2.75 Mb to 7.73 Mb) in the Taiwan indigenous populations of Ami and Atayal, encompassing numerous hemoglobin and olfactory receptor genes including the beta globin gene (*HBB*) that contains three nonsynonymous mutations (HbC, HbS, HbE) that impair red blood cell functions and cause anemia. The frequency of this extended haplotype was inferred to be between 10% and 15% in the Taiwan indigenous populations, and both characteristics of the haplotype length and frequency were similar to that of the malaria-driven selection signal in the HapMap2 Africans (YRI, frequency of 12.5% and length of haplotype present at top 0.1% of the genome-wide distribution [23, 28]). The HSI for the selected haplotype forms in YRI and the Taiwan populations was 0.63, suggesting that the two signals of positive selection were likely to have undergone convergent evolution and have emerged independently if the Taiwan selection signal was driven by genetic advantage to malaria resistance (Figure 7).

**Figure 8:**
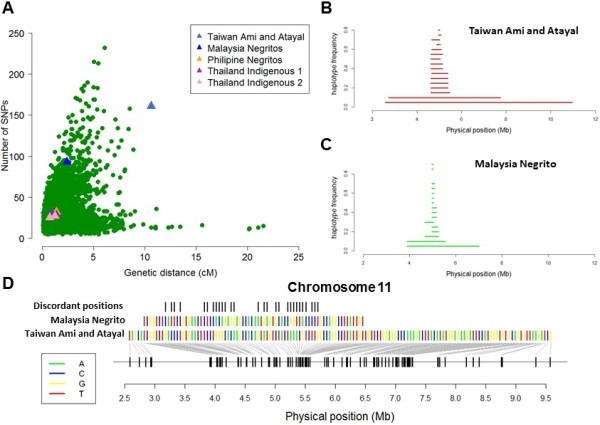
**Haplotype characteristics surrounding** ***HBB.*** **(A)** Scatterplot of the number of SNPs against the genetic distance spanned by the longest haplotypes found at 10% in five collections of indigenous populations in Asia, including two from Thailand and China (Indigenous 1: China Wa and Thailand H’Tin, Mlabri, Plang, Karen and Lawa ethnicities; Indigenous 2: Thailand Tai Lue, Tai Yong, Tai Kern and Tai Yuan ethnicities). The corresponding signals that span *HBB* in the five collections have been represented by triangles. **(B)** A stacked haplotype plot for Taiwan Ami and Atayal, where the longest haplotype around *HBB* at each core haplotype frequency from 5% to 95% in increments of 5% is illustrated. **(C)** A similar stacked haplotype plot around *HBB* for Malaysia Negrito. **(D)** A comparison of the longest haplotype forms around *HBB* present at 10% in Malaysia Negrito and Taiwan Ami and Atayal samples, where the discordant positions between the two haplotype forms are illustrated in the top panel.

As the Taiwan indigenous people are of Southeast Asian origins and they speak a language belonging to the Malay-Polynesian linguistic system that is related to Indonesia and the Philippines, we were interested to evaluate whether other indigenous populations from Southeast Asia exhibited similar evidence of positive selection that may have failed to reach the discovery threshold adopted by haploPS as a consequence of the lower SNP density. We observed that there were no evidence of uncharacteristically long haplotypes surrounding *HBB* in the Philippines Negrito and two groups of Thailand indigenous populations (Figure 8A, Additional file 1: Figure S3) as compared to that present in the Taiwan indigenous populations (Figure 8B), although there appeared to be extended haplotypes spanning in excess of 1.5 Mb in the Malaysian Negrito at frequencies of 10% and 5% (Figure 8C) which however did not meet the discovery criterion. The haplotype forms (at the haplotype frequency of 10%) in Malaysian Negrito and in Taiwan Ami and Atayal were discordant at numerous sites and had a HSI of 0.81 (Figure 8D). This indicated that even if there was genuinely a positive selection signal surrounding *HBB* in the Malaysian Negrito, this is likely to have happened independent of the evolutionary event in the Taiwan populations.

We also did a comparison of the selection regions identified by HaploPS and the top genes under selection detected by the Fst approach as reported in the previous study by Qian and colleagues [24]. We observed that of the 193 genes found by Fst, 57 were similarly present in 29 of the 59 regions identified by HaploPS (Additional file 1: Table S2). The overlapping genes include *PIK3R3*, which was among the strongest signals by Fst approach and was functionally related immune protection and signal transduction. *ERBB4* was also found to be under positive selection in non-African populations by iHS, XLR and XP-EHH in previous studies [29]. The MHC region on chromosome 6 was also detected by both Fst and HaploPS. The remaining 30 regions were discovered uniquely by HaploPS, and which included the *HBB* gene in Taiwan aboriginals. This shows that our investigation using the haplotype-based method provides additional evolutionary insights for the PASNP data, where it not only provided additional evidence for regions identified by Fst, but also discovered novel regions under positive selection.

## Discussion

Due to the sparse SNP density of the PASNP data, locating genomic signatures of positive selection has previously relied on a SNP-based approach that essentially prioritized genomic regions with significant differentiation in allele frequency to indicate the presence of positive selection. Here we have utilized haploPS, a haplotype-based method of detecting positive selection by explicitly characterizing the haplotype form that is carrying the advantageous variant, to identify evidence of positive selection in 31 groups of populations that have been clustered on the basis of genetic, linguistic and geographical similarities. Empirical comparison of the consistency in selection signals identified with the original and thinned SNP data from HapMap2 indicated haploPS had the highest specificity, and simulations indicated that the method was also effective at detecting selection signals at low frequencies in the populations. HaploPS successfully located 59 genomic regions undergoing positive selection that distinguished the aboriginal people from Malaysia, Philippines, Taiwan and China from the rest of the populations. Characterizing the inferred frequencies of the advantageous variants indicated that most of the low frequency signals were found in the ethnic minorities and indigenous populations, with urban and cosmopolitan populations being more likely to carry medium to high frequency signals.

Haplotype-based approaches to locate selection signals require: (i) accurate haplotype phasing across the SNPs to preserve the extended haplotype structure; and (ii) sufficient number of SNPs to be present on the extended haplotype to anchor the signal. In this paper, we have illustrated that the sparse SNP density provided a double whammy to the statistical power to locate selection signals, since the lower density also affected the accuracy of haplotype phasing by introducing more switch errors that can break up the structure of extended haplotypes. In light of this, we have focused on reporting only what have been observed to be positively selected, and the absence of classical signals such as the pigmentation-linked *KITLG* and East Asian hair morphology-linked *EDAR* is likely to be attributed to the lower power to identify genuine signals. One example that illustrates this is the skin pigmentation locus *ADAM17* that was found to be positively selected in all four East Asian populations (CHB, CHD, CHS, JPT) in HapMap and SGVP. Our analyses similarly identified this locus in the Japan Okinawans and Koreans, but failed to locate a signal in other East Asian populations.

Of the 59 regions identified in this analysis, 34 of them overlapped with positive selection signals previously reported in East and Southeast Asian populations from HapMap and SGVP, and where 24 of the signals were present in at least three of these populations [23]. However, the sparse SNP density meant that some of these regions spanned considerable distances and thus encompassed multiple signals discovered in the HapMap and SGVP datasets with almost 30-fold higher SNP density. Indeed one of the limitations of the current analysis is the over-estimation in the size of the genomic regions which will require data of higher SNP density in the corresponding populations to fine map. Interestingly, of the 25 signals that were present only in the PASNP dataset, 24 of them were found in ethnic minorities or aboriginal populations, raising the possibility that the majority of these signals were evidence of local adaptation found only in these indigenous groups.

The discovery of a positive selection signal that extends for almost 5 Mb around the cluster of hemoglobin genes (including *HBB*) in the Taiwan Ami and Atayal may present the first evidence of genetic resistance against malaria. The incidence of thalassemia is higher in the Taiwan indigenous populations than the urban populations, and there are at least two possible hypotheses: (i) malaria is endemic in Malaysia and the Philippines and based on existing evidence that suggests the Taiwan aborigines are related to the indigenous people of Malaysia and/or the Philippines, the advantageous mutations may have arisen prior to the divergence of the Taiwan and these Southeast Asian indigenous people; (ii) malaria is present in Taiwan and that has driven the emergence of genetic factors providing host resistance to malaria, in a similar situation as in African populations such as the Gambia, Nigeria and Kenya. The PASNP data included samples from Malaysian and Philippines Negrito, and our survey did not present any conclusive evidence of extended haplotypes in these populations, or any indication that the haplotypes from these Southeast Asian populations were similar to the selected haplotype form in the Ami and Atayal. There have however been historical reports of migrant Chinese being more susceptible to malaria than the Taiwan aborigines [30, 31] and evidence of malarial selection on specific immunoglobulin allotypes in the Taiwan aborigines [32], indicating that the second hypothesis is possible in light of the high infant mortality attributed to malaria in the absence of modern healthcare.

Population bottlenecks can reduce genetic variation in a population and the resultant homogeneity can be mistaken as evidence of positive selection. Whether indigenous populations in Asia had experienced strong population bottlenecks as those recently reported in native Americans is unclear [33], but it is important to acknowledge that bottlenecks or inbreeding can increase false discoveries of positive selection in a population. We observed that majority of the signals identified in the ethnic minorities or indigenous populations were less frequent in the populations, similar to previous observations of positive selection signals in African populations [23]. These signals may belong to very recent evolutionary events that have occurred in the population, or may actually correspond to balancing selection which prevented the derived allele from sweeping to fixation. Access to modern healthcare can mitigate the selective pressure of genetic factors in determining survival and reproductive advantages, and genetic adaptation is likely to be an ongoing process in indigenous populations due to the tendency to reside in natural habitats and rely on traditional medicine.

## Conclusions

This study has established that the lower SNP content of the PASNP data conferred weaker ability to detect signatures of positive selection, but the availability of the new approach haploPS retained modest power. Despite the analysis of 73 Asian populations, we have only identified 59 signals, highlighting the need for a comprehensive survey of Asian genomics with either microarray data of higher SNP density or population-level whole-genome sequencing such as that by the 1000 Genomes Project, in order to understand the subtle variations in local adaption between Asian populations due to climate, diet and environmental differences. Indeed in the study of population genetics, both the presence and absence of adaptation can be insightful, evident in classical signals at the lactase gene (*LCT*) and genes related to skin pigmentation (*KITLG*, *SLC24A5*). However, genetics studies involving Asian ethnic minorities and indigenous populations will require careful engagement of local communities, as the challenges of educating and obtaining informed consent from these communities are comparable to the situation in Africa. This may require the cooperation and knowledge transfer between genetic scientists across Asia, to share experiences and leadership in research ethics, data analyses and the principles of data sharing and ownership in order to develop a genomics research network in Asia.

## Methods

### Dataset

The dataset from the HUGO Pan-Asian SNP Consortium consists of 1,928 individuals from 73 Asian populations nd 2 non-Asian HapMap populations (Europeans CEU, Nigeria Africans YRI) that have been genotyped on the Affymetrix GeneChip HumanMapping 50 K Xba Array. A total of 54,794 SNPs passed quality control and were present in all the populations. The 73 Asian populations represent major, ethnic minority and indigenous populations from East Asia, Southeast Asia and South Asia. The sample size of each population ranges from 5 in Melanesian to 90 in the Koreans.

### Grouping of Asian populations

The 73 Asian populations were partitioned into 31 groups according to the maximum likelihood phylogenetic tree for the populations as described in the original PASNP publication [25]. Briefly, this used the genotype data for SNPs in the autosomal chromosomes to perform a maximum-likelihood inference of population similarity with the CONTML program in the PHYLIP package [34]. Branches with insufficient bootstrap support (defined as <50%) were merged, and populations that were found in the same major branch but were geographically and linguistically similar were also merged to increase the sample sizes during the analysis for signatures of positive natural selection.

### Haplotype phasing and accuracy evaluation

Haplotypes for each individual were estimated from the genotype data with SHAPEIT [35] using the reference haplotypes from 1,092 individuals in Phase 1 of the 1000 Genomes Project [36]. Phasing was performed on 54,787 shared SNPs across all 1,928 individuals from the 73 Asian and two non-Asian HapMap populations within the same batch runs, which did not consider the existence of different populations. To evaluate phasing accuracy, we repeated the haplotype phasing using genotype data in the four populations (CEU, CHB, JPT, YRI) in Phase 2 of the International HapMap Project (HapMap2) [3] in two settings: (i) with the full set of autosomal SNP data; (ii) with a reduced set of autosomal SNPs that have been thinned to reflect the SNP density similar to the Affymetrix 50 K Xba array. The thinning was done using the PASNP dataset as a template. SNP markers that were common to both the HapMap2 and PASNP datasets were first selected. If a SNP marker in PASNP dataset was missing in HapMap2, the nearest neighboring position was chosen to represent the position. The resultant haplotypes across the 22 chromosomes for these samples are compared against those from the HapMap which we considered as the benchmark, as these have been phased with PHASE [37] and incorporated pedigree information in inferring the haplotypes for CEU and YRI trios [38]. The quality of the phasing was quantified by the switch error, obtained by the ratio of the number of switches in the SHAPEIT haplotypes that were needed to recover the HapMap-phased haplotypes to the total number of heterozygote markers minus one across the genome in each individual. The switch error was calculated for every individual and subsequently averaged across all the individuals in each population.

### Detecting positive selection in PASNP groups

Three different haplotype-based methods (haploPS, iHS, XP-EHH) and one allele frequency based method (Fst) were used to detect genomic signatures of positive selection on the reduced set of SNPs from the populations in HapMap2. However, only haploPS was used in the analysis of the PASNP data. Population-average recombination rates were used by all three methods. We used the C++ software for haploPS, iHS and XP-EHH that were publicly available at http://www.statgen.nus.edu.sg/~haplops and http://hgdp.uchicago.edu/Software/.

HaploPS performs an explicit search for uncharacteristically long haplotypes in the genome that are found at a particular frequency [23]. By performing an exhaustive search across the SNPs, haploPS quantifies the evidence of a long haplotype on the basis of the genetic distance (in cM) spanned and the number of SNPs that is present on the haplotype. Each of these haplotypes is assigned two empirical p-values, defined as: (i) the proportion of haplotypes across the genome that span a genetic distance at least as large as the candidate haplotype; (ii) the proportion of haplotypes that span as many SNPs as the candidate haplotype. These two empirical p-values are used to construct the haploPS score, defined as the product of the two empirical p-values multiplied by the total number of haplotypes across the genome. Note that the haploPS score does not have the interpretation of a traditional p-value, and can be larger than 1. Haplotypes with haploPS score < 0.05 are deemed to exhibit evidence of positive selection. This procedure is performed for each population grouping across a range of haplotype frequencies from 0.05 to 0.95, at a step-size increment of 0.05. At each genomic location, the significant haplotype found at the highest haplotype frequency is reported, and the estimated frequency of the advantageous allele is taken as the highest haplotype frequency where the haploPS score is significant.

The integrated haplotype score (iHS) was calculated by estimating the extended haplotype homozygosity (EHH) score. The EHH is the probability of identity-by-descent for two haplotypes that carry a core haplotype within a distance to a pre-defined focal SNP [39], and the iHS is the integration of EHH scores up to the SNP with an EHH score of 0.05, or until there is a gap of more than 2.5 Mb [8]. The iHS statistic can be artificially inflated in the presence of gaps ranging from 20 kb to 200 kb, and the statistic is corrected by a scaling factor according to that described by Voight and colleagues [8]. The raw iHS statistics are normalized within 20 derived allele frequency bins, and SNPs are subsequently grouped into non-overlapping windows of 1 Mb. The proportion of SNPs in each window with |iHS| > 2 is calculated, and windows with a degree of over-representation as found in the top 1% of all the windows are considered as candidate regions of positive selection.

The cross-population extended haplotype homozygosity (XP-EHH) contrasts evidence of positive selection between a target population and a reference population at a focal SNP [13]. At each focal SNP position, neighboring SNPs that are present in both populations and within 1 Mb of the focal SNP are used to calculate the XP-EHH score, provided there is at least one SNP in the region with an EHH between 0.03 and 0.05. A SNP with EHH nearest to 0.04 is identified, and the EHH scores across all the SNPs between the focal SNP and the identified SNP are integrated. The XP-EHH statistic is defined as the logarithm of the ratio of this integral in the target population with respect to the reference population. The genome-wide distribution of the raw XP-EHH statistics is standardized to zero mean and unit variance. SNPs are subsequently grouped into non-overlapping windows of 1 Mb, and the maximum XP-EHH score in each window is denoted. Candidate selection regions are identified as windows found in the top 1% of the distribution of the maximum XP-EHH scores. We used YRI as the reference population for all non-African population groups, and CEU was used as the reference population for YRI.

Fst analysis was performed following description of Qian et al. [24]. For each pair of populations, we calculated the Weir and Hill unbiased Fst on the common set of SNPs. Then a sliding window approach was used, where the window size is set to be 500 Kb. In each window, the average value of the highest three Fst values were used as the window’s statistics. Windows with smaller than five SNPs were excluded from the analysis. The significance threshold was defined as top 1% and windows with statistics larger than the threshold were considered as regions with positive selection.

### Power simulation for haploPS at a reduced SNP density

In order to assess the degree of power loss of haploPS at a reduced SNP density similar to that of the PASNP data, we utilized the simulation data for haploPS that have been made publicly available at http://www.statgen.nus.edu.sg/~haplops/ and thinned the available dataset to 1/20^th^ of the original SNP density. Briefly, the dataset has been simulated using *SelSim*[40] which produced genotype data for a region undergoing positive selection by introducing an advantageous mutation at a pre-specified location. The selection coefficient was set as 0.01, and the frequency of the advantageous mutation was set to range between 10% and 100%, in increments of 10%. The effective population size N_e_ was assumed to be 17,469, and the mutation rate was set to 3 × 10^−8^ per base per generation. The recombination rate was generated by *cosi*[41] with a baseline rate of 1 cM/Mb. The simulation was performed to generate SNPs in 100 kb regions, where the original simulations yielded an average of 200 SNPs per 100 kb (a SNP density similar to that present in HapMap Phase 2) although we thinned the density to 1/20^th^ of the original density (or around 10 SNPs per 100 kb) to reflect the SNP density that is present in the PASNP data. Null simulations were performed with *cosi* to generate regions without positive selection. A total of 2,000 positively selected regions and 2,000 neutral regions were generated. HaploPS was applied to both the original simulated dataset and the reduced-density dataset to evaluate statistical power. The power is quantified as the fraction of the 2,000 positive selection iterations where the haploPS score obtained is less than the 1^st^ percentile of the distribution of haploPS scores obtained from the 2,000 iterations under the null model.

### Population clustering of positive selection signals

For the 59 genomic regions that have been identified to be positively selected in at least one of the 31 population groupings, we constructed a 31 × 59 indicator matrix, where the (*i*, *j*)th element of the matrix takes value 1 if the *j*th region is found to be positively selected in population *i*, and is 0 otherwise. This is used to calculate a 31 × 31 correlation matrix, which indicates the degree of sharing of the 59 selection signals across the 31 population groupings. This correlation matrix is used to perform a hierarchical clustering using the Ward’s minimum variance method implemented in *hclust* in R.

### Quantifying haplotype similarity and inferring origin of shared selection events

To infer whether positive selection events shared by multiple populations originated from the same mutation event or from separate mutation events, we assessed the degree of similarity between the identified haplotype forms in the different populations using the haplotype similarity index (HSI) [23]. This assumes that if the advantageous mutation arises before the different populations diverged, then the same haplotype form will be identified to carry the advantageous allele across the different populations and we will thus expect a significant degree of similarity in the identified haplotype forms. Conversely, if the locus is positively selected in multiple populations due to independent emergence of the same or different advantageous alleles in the locus, these alleles would have arisen on different haplotypes and thus the identified haplotype forms will be considerably different. Thus, for a region that is found to be positively selected by haploPS in *K* populations, we can identify the *K* selected haplotype forms for these populations respectively, and compare the alleles at the common set of *L* SNPs. The *K* × *K* similarity matrix *M* is calculated such that the leading diagonal entries are all ones, and the (*i*, *j*)^th^ entry of the matrix corresponds to the scaled Manhattan distance between the selected haplotype forms for population *i* and population *j* defined as *M*(*i*, *j*) = 1–*l*/*L* with *l* represent the number of sites out of *L* where the two haplotypes carry different alleles. An eigen-decomposition is performed on the matrix *M*, and the haplotype similarity index (HSI) is defined as the amount of variance explained by the first principal component. We infer a shared signal as a single mutation event if the HSI > 0.98, and as convergent evolution if HSI < 0.9.

## Electronic supplementary material

Additional file 1: **Supplementary material.** Detecting evidence of positive selection across Asia with sparse genotype data from the HUGO Pan-Asian SNP Consortium. (DOCX 338 KB)
