# Supplementary material for: Evaluating the possibility of detecting evidence of positive selection across Asia with sparse genotype data from the HUGO Pan-Asian SNP Consortium
Source: BMC Genomics. 2014 May 2;15(1):332. doi: 10.1186/1471-2164-15-332 (PMC4035063; doi:10.1186/1471-2164-15-332)
Supplement: Supplementary file 1 — Additional file 1: Supplementary material. Detecting evidence of positive selection across Asia with sparse genotype data from the HUGO Pan-Asian SNP Consortium. (DOCX 338 KB) [file 12864_2013_6034_MOESM1_ESM.docx]

**SUPPLEMENTARY MATERIAL**

**Detecting evidence of positive selection across Asia
with sparse genotype data from the
HUGO Pan-Asian SNP Consortium**

**Xuanyao Liu, Woei-Yuh Saw, Mohammad Ali, Rick Twee-Hee Ong, Yik-Ying Teo**

**CONTENTS**

**1 Supplmentary Methods 2**

1.1 Quantifying over-representation of height genes 2

**2 Supplementary figures 3**

**3 Supplementary tables 6**

**4 References 10**

**1 Supplementary Methods**

**1.1 Quantifying over-representation of height genes**

Of the 59 genomic regions identified by haploPS to be positively selected in the 31 PASNP population groupings, 30 regions containing a total of 3,518 genes were found to possess at least one height-associated gene. Given that there have been more genome-wide association studies (GWAS) in height, particularly those involving hundreds of thousands of samples, we wanted to evaluate whether there was any evidence of over-representation for positive selection in height-related genes in the PASNP populations. As of 24 June 2013, there were 279 genes reported to be associated with height in the NHGRI GWAS catalogue[^1^](#_ENREF_1), against a baseline of 28,906 genes in the autosomal chromosomes of the human genome. The 30 positively selected regions contained 58 height-related genes, and a one-sided Binomial test yielded a p-value of 9.98 × 10^-5^ that this observation was due to chance.

**2 Supplementary figures**

**Supplementary Figure 1. Evidence of positive selection by iHS in HGDP populations**

**
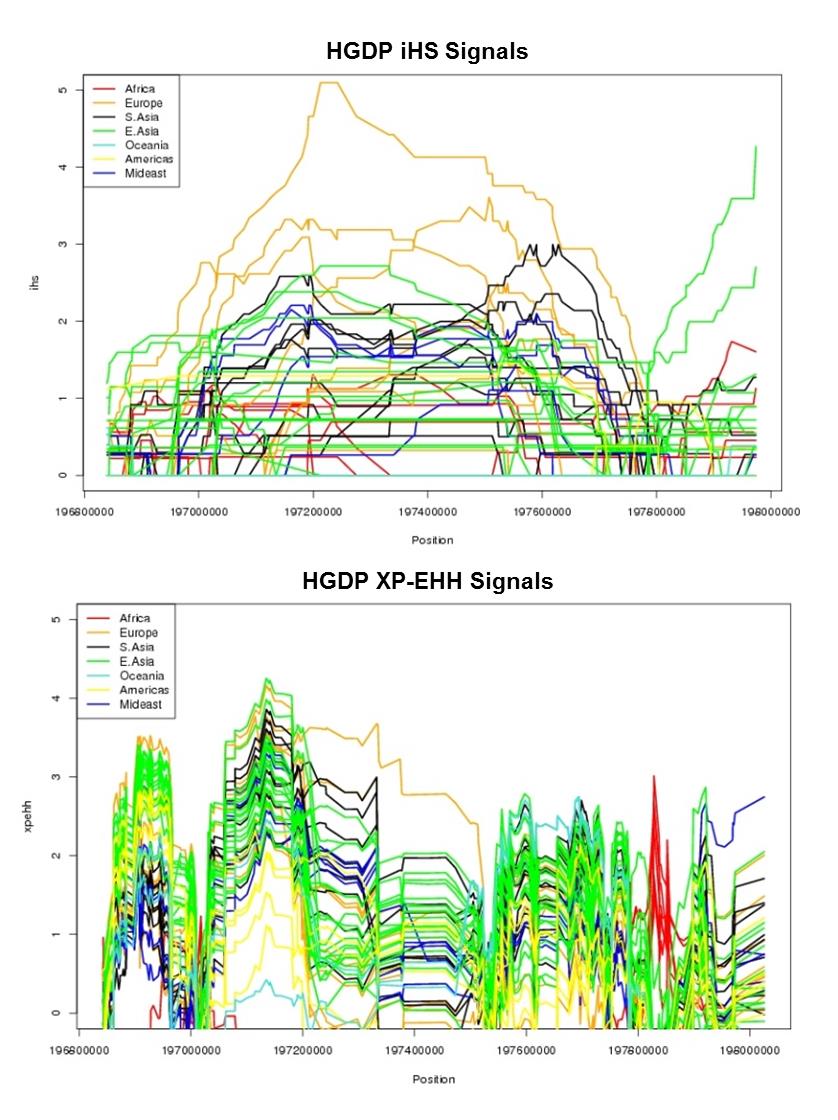
**

Evidence of positive selection by iHS and XP-EHH for the populations in the Human Genome Diversity Project at chromosome 2. The figures were obtained from the HGDP Selection Browser maintained by the Pritchard Lab at <http://hgdp.uchicago.edu/cgi-bin/gbrowse/HGDP/> using input coordinates of Chr2:196,841,741..197,997,071.

**Supplementary Figure 2. Selected haplotype forms in 12 PASNP population groupings**

**
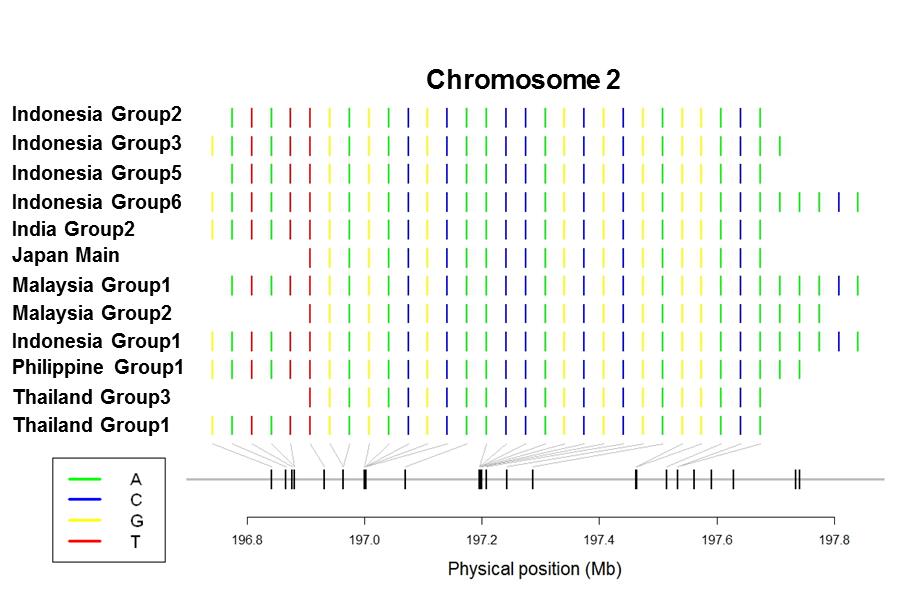
**

HaploPS identified the extended haplotypes that presented evidence of positive selection at chromosome 2 between 196.8Mb and 198.0Mb in 12 of the 31 PASNP population groupings. By extracting the haplotypes at frequencies ranging between 45% and 85% in the respective populations, we can infer that the selection signals likely stem from the same evolutionary event prior to the divergence of the populations as the selected haplotypes were perfectly identical and yielded a haplotype similarity index (HSI) of 1.00.

**Supplementary Figure 3. HaploPS evidence around the *HBB* locus**

**
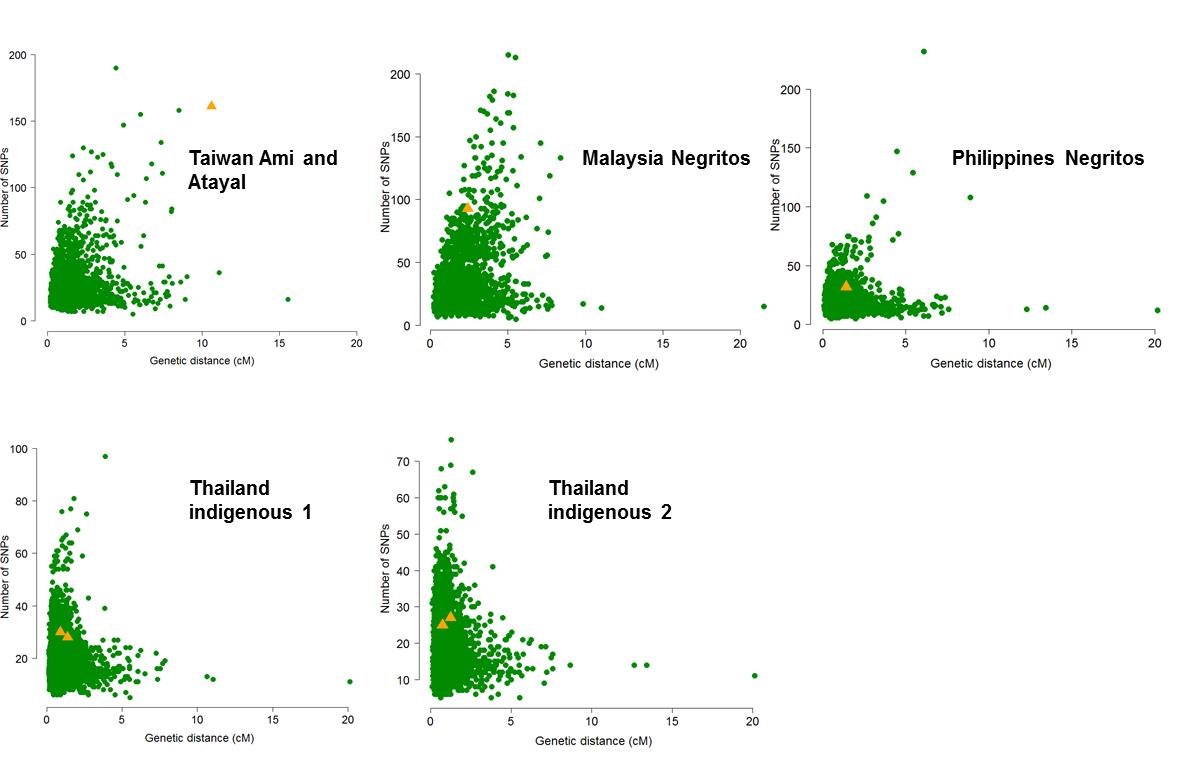
**

The horizontal axis of each panel shows the genetic distance in centimorgans spanned by the longest haplotype at 10% frequency across the genome, while the vertical axis shows the number of SNPs spanned by the corresponding haplotype. Thailand indigenous 1 refers to the PASNP populations from Thailand of H’Tin, Mlabri, Plang, Karen and Lawa ethnicities and the China Wa ethnic group; while Thailand indigenous 2 refers to the PASNP populations from Thailand of Tai Lue, Tai Yong, Tai Kern and Tai Yuan ethnicities.

**3 Supplementary tables**

**Supplementary Table 1. Labeling and characteristics of the populations in PASNP.** This table is adapted from Figure 1 in the original PASNP publication[^2^](#_ENREF_2).

| **Grouping** | **Labels** | **Country** | **Ethnicity** | **Language** | **# samples** |
| --- | --- | --- | --- | --- | --- |
| China Group 1 | CN-GA | China | Han | Cantonese | 30 |
| China Group 2 | CN-HM | China | Hmong | Hmong | 26 |
| China Group 3 | CN-JI | China | Jiamao | Jiamao | 31 |
|  | CN-CC | China | Zhuang | Zhuang | 26 |
| China Group 4 | CN-UG | China | Uyghur | Uyghur | 26 |
| China Han | CN-SH | China | Han | Chinese | 21 |
|  | CHB | China | Han | Chinese | 45 |
| Indonesia Group 1 | ID-SB | Indonesia | Kambera | Kambera | 20 |
|  | ID-RA | Indonesia | Manggarai | Manggarai | 17 |
|  | ID-SO | Indonesia | Manggarai | Manggarai | 19 |
|  | ID-LA | Indonesia | Lamaholot | Lamaholot | 20 |
|  | ID-LE | Indonesia | Lembata | Lembata | 19 |
|  | ID-AL | Indonesia | Alorese | Alor | 19 |
|  | AX-ME | Pacific | Melanesian | Nasioi | 5 |
| Indonesia Group 2 | ID-TR | Indonesia | Toraja | Toraja | 20 |
|  | ID-MT | Indoensia | Mentawai | Mentawai | 15 |
| Indonesia Group 3 | ID-ML | Indonesia | Malay | Malay | 12 |
| Indonesia Group 4 | ID-KR | Indonesia | Batak Karo | Batak Karo | 17 |
|  | ID-TB | Indonesia | Batak | Batak Toba | 20 |
| Indonesia Group 5 | ID-DY | Indonesia | Dayak | Benuak | 12 |
| Indonesia Group 6 | ID-SU | Indonesia | Sudanese | Sunda | 25 |
|  | ID-JA | Indonesia | Javanese | Javanese | 34 |
|  | ID-JV | Indonesia | Javanese | Javanese | 19 |
|  | MY-BD | Malaysia | Bidayuh | Jagoi | 50 |
| India Group 1 | IN-NI | India | Tharu | Pahari | 20 |
| India Group 2 | IN-TB | India | Ladakhi | Spiti | 23 |
| India Group 3 | IN-DR | India | Upper Caste | Telugu | 24 |
|  | SG-ID | Singapore | India Origin | Tamil | 30 |
| India Group 4 | IN-WI | India | Bhil | Bhili | 25 |
|  | IN-EL | India | Upper Caste | Bengali | 16 |
|  | IN-SP | India | Upper Caste | Hindi | 23 |
|  | IN-WL | India | Upper Caste | Marathi | 14 |
|  | IN-IL | India | Upper Caste | Hindi | 15 |
|  | IN-NL | India | Upper Caste | Hindi | 15 |
| Japan Main | JP-ML | Japan | Japanese | Japanese | 71 |
|  | JPT | Japan | Japanese | Japanese | 44 |
| Japan Okinawa | JP-RK | Japan | Ryukyuan | Okinawan | 49 |
| Korean | KR-KR | Korea | Korean | Korean | 90 |
| Malaysia Group 1 | MY-KN | Malaysia | Malay | Malay | 30 |
|  | MY-MN | Malaysia | Malay | Minangkabau | 20 |
|  | SG-MY | Singapore | Malay | Malay | 18 |
| Malaysia Group 2 | MY-TM | Malaysia | Proto-Malay | Temuan | 49 |
| Malaysia Negrito | MY-JH | Malaysia | Negrito | Jehai | 50 |
|  | MY-KS | Malaysia | Negrito | Kensiu | 30 |
| Philippines Group 1 | PI-MA | Philippines | Manobo | Manobo | 18 |
|  | PI-UI | Philippines | Urban | Visaya | 20 |
|  | PI-UN | Philippines | Urban | Tagalog | 19 |
|  | PI-UB | Philippines | Urban | Ilocano | 20 |
|  |  |  |  |  |  |
|  |  |  |  |  |  |
|  |  |  |  |  |  |
| ***Grouping*** | ***Labels*** | ***Country*** | ***Ethnicity*** | ***Language*** | ***# samples*** |
| Philippines Negrito | PI-AT | Philippines | Negrito | Ati | 23 |
|  | PI-IR | Philippines | Negrito | Iraya | 9 |
|  | PI-MW | Philippines | Negrito | Mamanwa | 19 |
|  | PI-AG | Philippines | Negrito | Agta | 8 |
|  | PI-AE | Philippines | Negrito | Aeta | 8 |
| Singapore Chinese | SG-CH | Singapore | Han | MinNan | 30 |
| Taiwan Indigenous | AX-AM | Taiwan | Ami | Ami | 10 |
|  | AX-AT | Taiwan | Atayal | Atayal | 10 |
| Taiwan Main | TW-HA | Taiwan | Han | Hakka | 32 |
|  | TW-YA | Taiwan | Han | MinNan | 48 |
| Thailand Group 1 | TH-HM | Thailand | Hmong | Hmong | 20 |
|  | TH-YA | Thailand | Yao | Iu-Mien | 19 |
| Thailand Group 2 | TH-TY | Thailand | Tai Yong | Tai Yong | 18 |
|  | TH-TL | Thailand | Tai Lue | Lue | 20 |
|  | TH-TK | Thailand | Tai Kern | Tai Kern | 18 |
|  | TH-TU | Thailand | Tai Yuan | Tai Yuan | 20 |
| Thailand Group 3 | TH-TN | Thailand | H’Tin | Mal | 18 |
|  | TH-MA | Thailand | Mlabri | Mlabri | 18 |
|  | TH-PP | Thailand | Plang | Blang | 18 |
|  | TH-KA | Thailand | Karen | Karen | 20 |
|  | TH-LW | Thailand | Lawa | Lawa | 19 |
|  | CN-WA | China | Wa | Wa | 56 |
| Thailand Group 4 | TH-PL | Thailand | Palong | Palong | 18 |
|  | CN-JN | China | Jinuo | Jinuo | 29 |
| Thailand Group 5 | TH-MO | Thailand | Mon | Mon | 19 |

**Supplementary Table 2. Regions identified by haploPS to be positively selected in the 31 PASNP population groupings.** The start and end coordinates for each region are reported in NCBI Build 36 coordinates.

**Supplementary Table 2 continued.**

**4 References**

1. Hindorff, L.A. *et al.* Potential etiologic and functional implications of genome-wide association loci for human diseases and traits. *Proc Natl Acad Sci U S A* **106**, 9362-7 (2009).

2. Abdulla, M.A. *et al.* Mapping human genetic diversity in Asia. *Science* **326**, 1541-5 (2009).
